# Long-Term Safety of PEGylated Coagulation Factor VIII in the Immune-Deficient Rowett Nude Rat

**DOI:** 10.1155/2017/8496246

**Published:** 2017-03-28

**Authors:** Caroline E. Rasmussen, Jette Nowak, Julie M. Larsen, Emma Moore, David Bell, Kai Chiu Liu, Nanna Skall Sorensen, Wendela A. Kappers, Thomas Krogh-Meibom, Hanne Offenberg

**Affiliations:** ^1^Non-Clinical Development, Novo Nordisk A/S, Måløv, Denmark; ^2^Envigo, Huntingdon, UK

## Abstract

Turoctocog alfa pegol (N8-GP) is a glycoPEGylated human recombinant factor VIII for the treatment of hemophilia A. The safety profile of rFVIII, and polyethylene glycols (PEG) technology, is well-established. Conducting long-term toxicity studies in animals using human proteins can be complicated by anti-drug antibody (ADA) development. To evaluate long-term safety of N8-GP, 26- and 52-week toxicity studies were conducted in immune-deficient rats dosed intravenously every fourth day with 0, 50, 150, 500, or 1200 IU/kg N8-GP. Observations included clinical observations, body weight, ophthalmoscopy, hematology, chemistry, coagulation, urinalysis, toxicokinetics, antibody analysis, and macroscopic/microscopic organ examination. Immunohistochemical staining examined the distribution of PEG in the brain. No adverse test item-related findings were seen and PEG was not detected in the brain. Exposure was confirmed for ~75% of the animals dosed with 500 and 1200 IU/kg N8-GP; the high lower limit of quantification of the bioanalysis assay prevented confirmation of exposure in the lower doses. A small number of animals developed ADAs, and the proportion of animals surviving until scheduled termination was >80%. N8-GP was well tolerated, and the immune-deficient rat proved suitable for testing long-term toxicity of human proteins that are immunogenic in animals.

## 1. Introduction

Replacement therapy with intravenously delivered plasma-derived or recombinant factor VIII (rFVIII) concentrates is the cornerstone for managing hemophilia A. Prophylactic FVIII replacement is preferred to on-demand therapy because it reduces the frequency of bleeds and prevents the development of chronic arthropathy in the long term. Currently available rFVIII concentrates with a prolonged plasma half-life necessitate intravenous injection at least every four days for prophylaxis [1]. Turoctocog alfa pegol (N8-GP, Novo Nordisk A/S, Måløv, Denmark) is a novel, glycoPEGylated, extended half-life rFVIII product in development for the prophylaxis and treatment of bleeding episodes in patients with hemophilia A. Attachment of a 40 kDa branched polyethylene glycol (PEG) molecule to rFVIII extends the half-life so that higher trough levels of native FVIII are available to provide safe and effective prophylaxis. PEGylation is a well-established protraction technology and it is used in more than 10 licensed products to extend the circulating half-life of proteins [2]. However, the knowledge of the long-term use of 40 kDa PEG is limited [3], and vacuole formation in various tissues has been reported in animals dosed with PEGylated compounds [4–7].

The nonclinical safety evaluation of new therapeutic drugs should include a repeat-dose toxicity study of six months' duration in rodents, according to the International Conference on Harmonisation of Technical Requirements for Registration of Pharmaceuticals for Human Use guidelines [8, 9]. Human coagulation factors are highly immunogenic in animals, and this can result in the development of anti-drug antibodies (ADAs). ADAs to a human coagulation factor can alter the clearance and/or neutralize the pharmacological effect of the dosed coagulation factor [10–12]. Furthermore, ADAs cross-reacting with the animals' endogenous coagulation factors may result in a state of acquired hemophilia in the animals [13]. This immune response precludes the possibility of conducting chronic toxicity studies in normal healthy animals.

In order to perform long-term exposure studies with N8-GP without interference from ADAs, an immune-deficient rat model was selected. The Rowett nude rat (Crl:NIH-*Foxn1*^*rnu*^) has no thymus and therefore does not raise a T-cell-dependent antibody response against foreign proteins [14]. Owing to a lack of an efficient immune response, these animals must be housed under sterile conditions to minimize their exposure to pathogens. Under normal conditions, the Rowett nude rat would be expected to live for only four months, but when housed in a sterile environment its lifespan may be increased to two years [15].

The purpose of the current studies was to evaluate the long-term toxicity of N8-GP when administered intravenously once every fourth day to Rowett nude rats for 52 weeks (followed by a 12-week recovery period). An immunohistochemical (IHC) staining method specific for PEG was used to examine whether PEG was present in the choroid plexus and was selected as a primary tissue for this investigation based on reports that choroid plexus epithelial cells are a target for 40 kDa PEG distribution [4, 16, 17]. Also, 40 kDa PEG has been shown to be associated with the formation of vacuoles [3, 5].

## 2. Materials and Methods

### 2.1. Experimental Design

Two long-term toxicity studies in Rowett nude rats (Crl:NIH-*Foxn1*^*rnu*^), compliant with good laboratory practice, were conducted at Envigo, Huntingdon, UK, and sponsored by Novo Nordisk A/S. Formulation analysis, toxicokinetic analysis and evaluation, antibody analysis, and IHC were performed at Novo Nordisk A/S, Måløv, Denmark. The in-life phases of the studies were conducted between April 2013 and August 2014. The first study (hereafter referred to as the 26-week study) included a 26-week dosing period (dosing phase) followed by 26 weeks without administration of test item to assess recovery from any potential adverse events (recovery phase). The second study (hereafter referred to as the 52-week study) included a 52-week dosing period (dosing phase) followed by a 12-week recovery phase. The study designs are shown in Table 1. The 26-week duration was chosen since this study duration meets the regulatory requirements of a chronic toxicity study. The 52-week duration was selected to evaluate any potential findings related to PEGylation, as this has been described for other PEGylated compounds [5]. The dose levels were selected to cover the anticipated clinical dose (50 IU/kg) and multiples thereof: 150 (×3), 500 (×10), and 1200 (×24) IU/kg. The in-life experimental procedures were in accordance with provisions of the UK Animals (Scientific Procedures) Act 1986 (the Act) under an appropriate project animal license number.

### 2.2. N8-GP PEGylation

The PEGylation process [18] and preparation of rFVIII [19] have been described elsewhere.

### 2.3. Animals and Housing

The Rowett nude rats (Crl:NIH-*Foxn1*^*rnu*^) were supplied by Charles River (USA and Germany) and were acclimatized for ≥12 days before dosing began. Upon arrival, all animals were randomly allocated into groups of separate sexes. To minimize infection, the animals were housed in a full-barrier rodent facility and in sterilized cages. Moreover, their diet was irradiated, and their bedding, environmental enrichment, and water were sterilized. To reduce infection risk further, animals from the United States and Germany were not housed together in the same cages. At the start of dosing, animals in the 26-week study were aged 7–11 weeks (males weighing 216–380 g and females 156–242 g); those in the 52-week study were aged 8–16 weeks (males weighing 221–395 g and females 159–240 g).

### 2.4. Administration of Test Substance

Intravenous bolus injection was performed in all animals every fourth day, alternating between the left and right caudal veins. The dose was calculated from the most recently recorded body weight and volume, and dose volumes ranged from 1 to 5 mL/kg.

### 2.5. In-Life Observations

Animals were visually inspected for evidence of ill health or reaction to test item at least twice daily on nondosing days and at least five times daily on dosing days. Any deviation from normal was recorded at the time with regard to nature and severity, date and time of onset, and duration and progress of the observed condition, as appropriate. Injection sites were inspected daily and all animals had at least a weekly physical examination. Body weight and food consumption were recorded weekly. Ophthalmoscopy was performed with a binocular indirect ophthalmoscope using Mydriacyl® (Alcon Laboratories, Inc., Fort Worth, Texas, USA) to dilate the pupils. The eyes were examined before treatment in all animals in both studies and during the last week of the dosing phases in all main-phase animals of Groups One and Five.

Blood was sampled for toxicokinetic evaluation, antibody analysis, and clinical pathology from the sublingual vein, while the animals were anesthetized using isoflurane. Urine was collected over ~16 hours while the animals were individually housed overnight in a sterilized metabolism cage. The sampling schedule for toxicokinetic evaluation, antibody analysis, and clinical pathology is shown in Table 2.

### 2.6. Clinical Pathology

Clinical pathology parameters were clinical chemistry, hematology, coagulation, and urinalysis. Blood samples for clinical chemistry were collected in lithium heparin tubes and plasma was stored at ambient temperature until analysis. Blood samples for hematology were stabilized by ethylenediaminetetraacetic acid (EDTA) and stored at an ambient temperature until whole-blood analysis was performed. Blood samples for coagulation were stabilized by citrate (3.2%) and also stored at an ambient temperature until whole-blood analysis was performed. Clinical chemistry parameters comprised alkaline phosphatase, alanine aminotransferase, aspartate aminotransferase, creatinine kinase, total bilirubin, urea, creatinine, glucose, total cholesterol, triglycerides, sodium (Na), potassium (K), chloride (Cl), calcium, inorganic phosphorus, total protein, and albumin. Hematology included hematocrit, hemoglobin concentration, erythrocyte count, absolute reticulocyte count, mean cell hemoglobin, mean cell hemoglobin concentration, mean cell volume, red cell distribution width, total leucocyte count, differential leucocyte count, and platelet count. Coagulation parameters included activated partial thromboplastin time (aPTT) and prothrombin time. Blood samples were collected ~12 hours after dosing. Urine was examined for volume, appearance, pH, specific gravity, glucose, ketones, bilirubin, protein, Na, K, and Cl, and any sediment was examined using a microscope. Hematology was measured using a Bayer ADVIA® 120 apparatus (Siemens Healthcare GmbH, Erlangen, Germany), coagulation using an ACL™ series analyzer (Instrumentation Laboratory, Beckman Coulter, Miami Lakes, Florida, USA), and clinical chemistry using a Roche P Modular apparatus (Roche Diagnostics North America, Indianapolis, Indiana, USA), and urinalysis was conducted using a CLINITEK®500 MULTISTIX® (Siemens Healthcare GmbH, Erlangen, Germany) and Roche P Modular apparatus.

### 2.7. Antibody Analysis

Blood samples for antibody analysis were taken before dose at the appropriate time to minimize drug interference with the assay. A radioimmunoassay was used to detect anti-N8-GP antibodies in the citrated plasma samples in both studies. In brief, anti-N8-GP antibodies in animal samples were bound to radioactively labeled N8-GP prepared by random labeling of tyrosine residues with I-125 to a specific radioactivity concentration of approximately 15 *µ*Ci/*µ*g. Subsequently, immunoglobulin and immune complexes were bound to protein G-Sepharose 4 and precipitated by centrifugation. The radioactivity in the precipitate was proportional to the amount of anti-N8-GP antibodies in the sample. Samples were mixed with rabbit polyclonal antibodies against immunoglobulin G (IgG), IgM, and IgA (an in-house product), to bridge IgG, IgM, and IgA bound to N8-GP to protein G-Sepharose, thus ensuring that IgM and IgA antibodies in the samples were also precipitated. A sheep polyclonal antibody directed against the factor VIII part of N8-GP (product number ab20946, Abcam PLC, Cambridge, UK) was used as a positive control. Results were expressed as a percentage of bound over total radioactivity. For each assay, a cut point value was calculated as the mean + [*t*(0.05; 1-sided; df) *∗* SD] of 18 plasma samples from untreated healthy Rowett nude rats. This method of calculation corresponds to approximately 5% false-positive samples in the assay. Samples with a result above the cut point value in the screening assay were reanalyzed in the presence of excess unlabeled N8-GP to confirm the specificity of the anti-N8-GP antibodies (confirmatory assay). Samples were negative if the screening result was less than or equal to the cut point. Samples were positive if the screening result was above the cut point and the result of the confirmatory assay fulfilled predefined criteria for positive samples. The work was carried out in accordance with internal departmental procedures and previous assay validation.

### 2.8. Bioanalysis

A one-stage aPTT-based activity assay (SynthASil, Instrumentation Laboratory) with FVIII-deficient human plasma was used to detect FVIII coagulation activity in the rat-citrated (3.8%) plasma samples. Clotting time was calculated from the rate of change in turbidity. Commercially available normal human plasma (Batch number 503231 [Siemens Healthcare GmbH, Marburg, Germany]) with known FVIII activity was used to form a calibration curve, and, from this, the clotting activity of unknown samples was derived. In both studies, a lower limit of quantification (LLOQ) was calculated as 2.50 IU/mL + the highest measured endogenous activity in the nondosed or predose study samples. Quality-control samples consisting of N8-GP spiked into FVIII-deficient plasma were included in all analytical runs.

### 2.9. Toxicokinetics and Exposure

A full toxicokinetic profile was taken after the first dose and at week 26 in the 26-week study and additionally at week 52 in the 52-week study. The profile included nine sampling time points (before dose and 0.25, one, four, eight, 12, 24, 48, and 96 hours after dose) using a sparse sampling schedule. To confirm continuous exposure throughout the studies, blood was also sampled from three animals in each group every four weeks and four hours after dosing.

Plasma concentration-time data were evaluated by noncompartmental analysis in Phoenix WinNonlin® version 6.2. build 6.2.0.495 (Pharsight®, St. Louis, Missouri, USA) from composite profiles of mean data (*n* = 3 per time point).

### 2.10. Necropsy and Histology

All animals (scheduled and unscheduled deaths) were subjected to a detailed necropsy at the end of each study (i.e., after the main study and recovery periods of the 26- and 52-week studies), including a full macroscopic evaluation, weighing of organs (scheduled deaths only), and microscopic examination of tissues listed in Table 3. Animals were killed using carbon dioxide asphyxiation with subsequent exsanguination in a sequence that allowed intergroup comparison.

Tissues from each animal, except for testes and eyes, were fixed appropriately ≥24 hours (10% neutral buffered formalin). Testes and eyes were fixed in modified Davidson's fluid and Davidson's fluid [20], respectively. Brain, including choroid plexus, was processed and evaluated for the presence of PEG by a previously described IHC method [21].

In the 26-week study, the scheduled termination after the dosing phase was performed two to four days after last dose and the scheduled termination after the recovery phase was performed 26 weeks after the last dose. In the 52-week study, the scheduled termination after the dosing phase was performed four to six days after last dose and the scheduled termination after the recovery phase was performed 12 weeks after the last dose.

### 2.11. Immunohistochemistry

Brain tissue (including the choroid plexus) was processed for the presence of PEG by a previously described IHC method [21].

### 2.12. Statistical Analysis

Statistical analyses were performed as previously described [21]. In brief, statistical analyses were conducted separately for males and females and carried out using the individual animal; the exception to this was food consumption, which was analyzed on a cage basis. Group mean, standard deviation, and percentage deviation from controls were calculated for body weight, food consumption, clinical pathology parameters, and organ weights. The following parameters were statistically analyzed separately at each time point to compare the following in each group versus controls: body weight gains and food consumption over appropriate study periods; clinical pathology parameters; and organ weights. An *F*1 test was used to analyze parametric data unless there were only two groups, in which case a *t*-test was used. Nonparametric data were analyzed using an *H*1 test; where there were only two groups, a Wilcoxon rank-sum test was used. For some clinical pathology parameters, >75% of the data were the same across groups and were therefore analyzed using Fisher's exact test.

## 3. Results

### 3.1. In-Life Observations

There were no test item-related findings in observations made during the in-life phase of either of the studies. Signs related to the dose injection procedure were seen in all groups in both studies. The signs included reddening, swelling, erythema, bruising, and raised areas of the tail at the site of injection.

Unscheduled animal deaths occurred in all groups in both studies and were not considered related to the test item due to the distribution among groups and the histopathological findings/cause of death (Table 4). The majority of these unscheduled deaths were considered related to nonspecific inflammatory processes and infections and were therefore consistent with findings expected in an immune-deficient animal vulnerable to infection. There were 11 unscheduled deaths in the 26-week study: eight during the dosing phase and three during the recovery phase. There were 45 unscheduled deaths in the 52-week study: 42 during the dosing phase and two during the recovery phase. The survival percentages were 95% for the 26-week study and 83% for the 52-week study.

### 3.2. Clinical Pathology

There were no test item-related adverse effects apparent on clinical chemistry or hematology parameters, or urinalysis, in either the 26- or the 52-week study. Differences between groups were only observed in relation to an expected shortening of the coagulation parameter aPTT. For the 26-week study, at weeks 13 and 26, there was a dose-related shortening of mean aPTT at all doses in males, although the shortening did not reach statistical significance in Group Two males (50 IU/kg) in week 26 (Table 5). Shortening of aPTT was also seen at week 13 for females receiving 1200 IU/kg and at dose levels of 150 and 500 IU/kg (although it was not statistically significant at these doses). No response was seen in females during week 26. After the 26-week recovery period, the aPTT was similar to controls in both males and females.

In the 52-week study, shortening of aPTT was observed in males and females receiving 500 and 1200 IU/kg, with a marginal shortening of aPTT also occurring in weeks 13, 30, and 48 in males and females receiving 150 IU/kg; statistical significance was attained for females in weeks 13 and 48 (Table 5). After the 12-week recovery phase, the mean aPTT in both the males and females previously receiving 1200 IU/kg was similar to that in control males and females.

### 3.3. Antibody Analysis

Anti-N8-GP antibodies were detected in five of 157 (3.2%) tested animals in the 26-week study and in seven of 183 (3.8%) tested animals in the 52-week study. Group distribution of animals that were positive for anti-N8-GP antibodies during the dosing phases can be found in Table 6. Anti-N8-GP antibodies were not detected in animals during either of the recovery phases.

### 3.4. Bioanalysis

The LLOQ for the 26- and 52-week studies were 6.51 and 6.17 IU/mL, respectively. The LLOQ was defined as 2.50 IU/mL plus the highest measured endogenous activity in each study. As a result of biological variability, the highest measured activity in the two studies differed, which explains the difference in LLOQ between the studies. The results in the quality-control samples demonstrated good assay precision and accuracy performance in both studies. The overall coefficients of variation were between 4.37% and 7.94% and the relative errors between −15.56% and 5.36%.

### 3.5. Toxicokinetics and Exposure

Systemic exposure to N8-GP above the LLOQ was confirmed in week 26 for Group Five (1200 IU/kg) in 13 of 14 male animals and in 10 of 15 female animals. For animals in Group Four (500 IU/kg), exposure was confirmed in week 26 for nine of 15 male animals and for four of 15 female animals. For the 150 IU/kg group, few samples were above the LLOQ, while for 50 IU/kg, all samples were below the LLOQ. For the 500 and 1200 IU/kg dose levels, exposure increased with the dose level. For the once-monthly four-hour postdose exposure samples (to confirm exposure continually throughout the study), taken between week 1 and week 26, zero to two of six samples in Group Three, four to six of six samples in Group Four, and five to six of six samples in Group Five were above the LLOQ. FVIII activity was below the LLOQ in all analyzed plasma samples from control animals. There was an indication for higher exposure in males than females at 26 weeks for area under the plasma concentration-time curve (AUC) and AUC from zero to 24 hours (AUC_0–24 h_), although data were limited.

**Figure 1:**
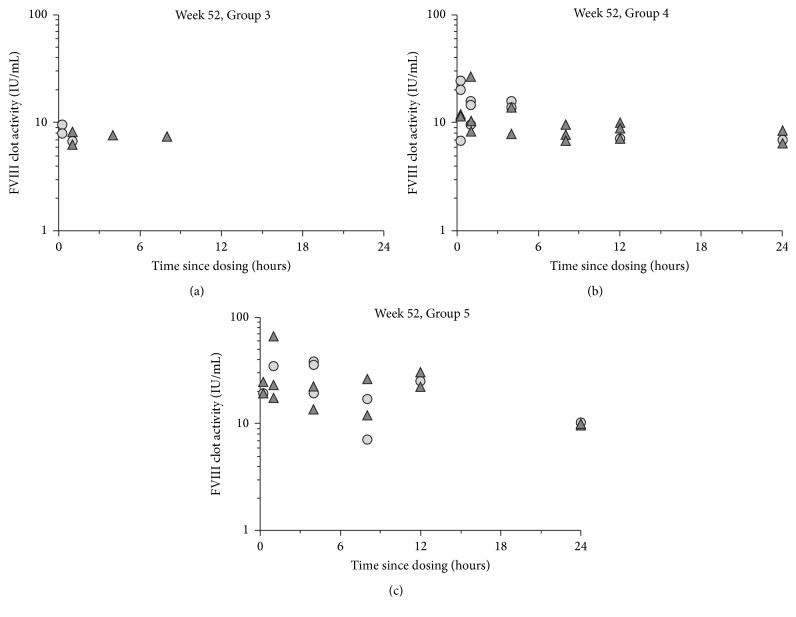
Individual FVIII clotting activity versus time in week 52 in male (triangles) and female (circles) Rowett nude rats following an intravenous dose of 150 IU/kg (Group 3), 500 IU/kg (Group 4), or 1200 IU/kg (Group 5) (sparse sampling with two samples per animal, in general* n* = 2-3 per sex per time point).

For the 52-week study, exposure to N8-GP above the LLOQ increased with dose (for plasma profiles, see Figure 1). Systemic exposure was confirmed in 13 of 15 males and in 10 of 15 females in Group Four (500 IU/kg), and in 14 of 15 males and in nine of 15 females in Group Five (1200 IU/kg). For the 150 IU/kg dose group (Group Three), only an occasional sample was above the LLOQ, and at 50 IU/kg (Group Two), all samples were below the LLOQ. Exposure increased with dose from 500 to 1200 IU/kg. During the once-monthly assessments (sampling at four hours after dose), none of six samples in Group Two (50 IU/kg), zero to two of six samples in Group Three (150 IU/kg), four to six of six samples in Group Four (500 IU/kg), and four to six of six samples in Group Five (1200 IU/kg) were above the LLOQ. There was a tendency for higher exposure in males than females for maximum observed plasma concentration (*C*_max_) and AUC_0–24 h_ in Group Five (1200 IU/kg) with *C*_max_ and AUC_0–24 h_ in males 43.6 IU/mL and 453 h*∗*IU/mL at day 1, and 36.3 IU/mL and 438 h*∗*IU/mL in week 52, respectively, while for females *C*_max_ and AUC_0–24 h_ were 28 IU/mL and 292 h*∗*IU/mL at day 1, and 31.4 IU/mL and 250 h*∗*IU/mL in week 52, respectively. This tendency was not apparent for Group Four (500 IU/kg). FVIII activity was below the LLOQ in all analyzed plasma samples from control animals, except one sample, which was slightly above the LLOQ.

### 3.6. Necropsy and Histology

There were no macroscopic or microscopic findings related to test item with N8-GP for up to 52 weeks' dosing or following the 12-week recovery period in previously treated animals.

### 3.7. Immunohistochemistry

**Figure 2:**
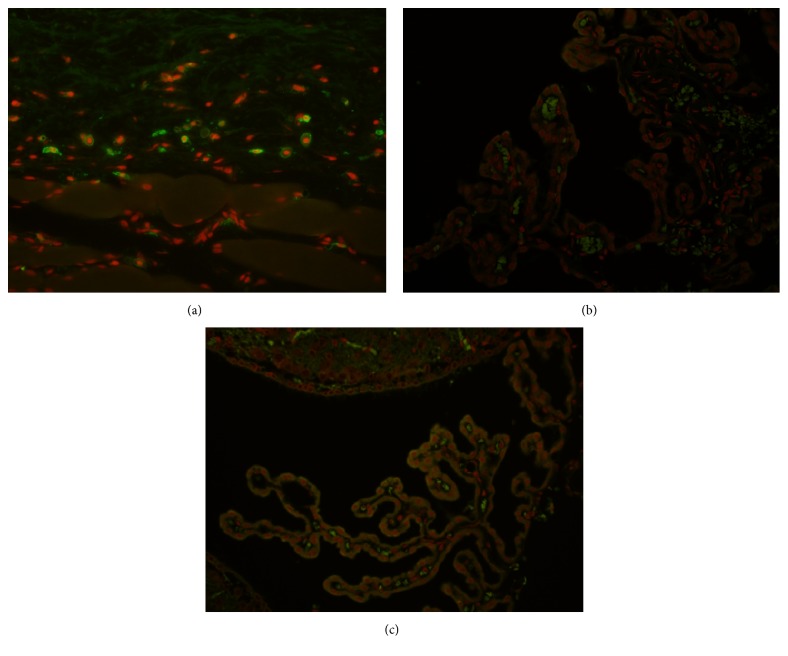
Immunohistochemical detection of polyethylene glycol (PEG) in (a) macrophages in subcutaneous tissue from a rat injected subcutaneously with 40 kDa PEG (positive control), (b) choroid plexus from rat dosed with 0 IU N8-GP/kg every fourth day for 52 weeks (negative control), and (c) choroid plexus from a rat dosed with 1200 IU N8-GP/kg every fourth day for 52 weeks. In (a), fluorescent green staining represents PEG content in macrophages. In (b) and (c), green staining represents autofluorescence staining of red blood cells in the core of the choroid plexus. Red staining in (a), (b), and (c) represents nuclei.

PEG was not detected in brain tissue (including the choroid plexus) or in blood in the brain blood vessels in either the 26- or 52-week study in main study animals dosed with 1200 IU/kg (Figure 2). IHC was not performed in the other dosing groups, or in recovery animals, because of the lack of positive IHC in the highest-dose animals.

## 4. Discussion

No test item-related adverse findings were observed at doses spanning 50 to 1200 IU/kg every fourth day in two toxicity studies of 26 and 52 weeks' duration in immune-deficient rats conducted to assess the long-term effects of 40 kDa PEGylation of human rFVIII. No PEG was detected in the choroid plexus using IHC staining and, in the absence of any adverse findings in these studies, the level of no observed adverse effect in Rowett nude rats was considered to be N8-GP 1200 IU/kg, dosed every fourth day.

The immune-deficient Rowett nude rat has been proven to be a suitable model for long-term toxicity studies in the current studies and in our previous study with N9-GP [21]. Although these results cannot be directly extrapolated to humans, it is worth noting that a study duration of 52 weeks in a rat model is equivalent to ~30 years in humans [22]. In both the 26- and 52-week studies, systemic exposure to N8-GP was confirmed for ~75% of the animals at 500 and 1200 IU/kg. For the lower dose levels, none or only an occasional sample was above the LLOQ at day 1 and at the end of the two studies. This was due to a high LLOQ, resulting from a combination of endogenous FVIII activity and matrix effects. The lack of confirmation of systemic exposure in the low-dose groups was not related to the model.

The survival percentage was higher in the 26-week study (95%) compared with the 52-week study (83%). Unscheduled deaths were observed to be evenly distributed among the N8-GP dosing groups. None of the unscheduled deaths in the current studies were considered to be related to test item. The animals that died prematurely showed signs of infection, such as urogenital and skin lesions, consistent with the lack of a functional immune system. This made them more vulnerable to infection, as would be expected in this rat model.

The Rowett nude rat was used because of its impaired ability to mount an immune response and hence generate ADAs that could alter the clearance of N8-GP and reduce N8-GP exposure. ADAs to N8-GP were detected in a few animals in both studies and were distributed evenly between the dosing groups. There is evidence that, in some T-cell-deficient nude rats, “T-like” cells develop increasing functionality with age [14], which is consistent with a few animals developing ADAs in these studies. However, since the frequencies were low and consistent between studies (i.e., 3.2% in the 26-week study and 3.8% in the 52-week study), the model still appears to be a valuable tool when long-term exposure studies without interfering ADA are needed. Thus, based on the high survival rate and limited number of animals developing ADAs, the nude rat animal model appears to be appropriate as a long-term toxicity model and could be considered as an alternative long-term rodent model to test human proteins immunogenic in animals. However, the survival rate and tendency for ADA development in a small percentage of animals should be considered when planning the duration of the study and the number of animals to be included. In addition, compounds with a known effect on the immune system may not be appropriate to test in this model due to the lack of a functional immune system.

Coagulation analyses demonstrated an expected shortening of aPTT in all dosed males in the 26-week study, and in males and females receiving 500 and 1200 IU/kg in the 52-week study. This effect is attributable to the pharmacological effect of N8-GP in these animals and was not considered an adverse finding. This interpretation is supported by the macroscopic and microscopic pathology data, where no findings were identified, which would otherwise indicate in vivo effects of the shortened aPTT. The lack of response in females at week 26 of the 26-week study could be explained by differences in postdose sampling time points; the time between dosing and sampling for the clinical pathological investigation was two to four days after dose for females at week 26, in contrast to 12 hours after dose for all other clinical pathology investigations. The tendency toward a higher exposure in males might explain the more prominent shortening of aPTT in males compared with females.

None of the toxicity endpoints (clinical observations, body weight gain and food consumption, ophthalmologic examination, clinical chemistry, hematology, and urinalysis) were affected by test item.

After 26 and 52 weeks of N8-GP dosing every fourth day, up to 1200 IU/kg, no test item-related vacuoles were observed in any of the hematoxylin and eosin- or IHC-stained tissues, and no PEG was detected in the brain or choroid plexus, or in the blood in the blood vessels of the brain. In contrast to the present studies with N8-GP, in which no PEG retention was observed, our previous study evaluating the long-term toxicity of N9-GP in immune-deficient athymic rats showed a dose-dependent increase in the presence of PEG in the choroid plexus epithelial cells over 26 weeks, although this did not lead to pathological changes or test item-related vacuole formation in these cells [21]. This discrepancy in PEG retention between the two molecules may be due to the smaller PEG load per unit/kg of N8-GP versus N9-GP: a dose of 50 IU/kg N8-GP corresponds to a PEG load of 1 *µ*g/kg [21], whereas 40 IU/kg N9-GP corresponds to 200 *µ*g/kg PEG [21]. There are two other PEGylated FVIII candidates indicated for the treatment of hemophilia A, developed by Bayer and Baxalta. Bayer's BAY 94-9027 is a B-domain-deleted rFVIII with site-specific PEGylation, and a 60 kDa branched PEG [23]. Baxalta's approved Adynovate® (BAX 855) is a full-length rFVIII, conjugated with 20 kDa branched PEG molecules, and is based on Advate® [24, 25]. Although long-term animal toxicology studies such as our current study have not been conducted on these proteins, 28-day studies in rats and monkeys identified no safety concerns associated with PEG at clinically relevant dose levels [23, 24].

The inability to provide proof of exposure at the lower dose levels is a limitation to this study. However, considering that no test item-related findings were seen at any dose level and exposure was confirmed at high dose levels, this limitation is not believed to impact the overall interpretation of the study. Another limitation is the timing of blood sampling from females at week 26 of the 26-week study, which was postponed from 12 hours to two to four days after last dose. Appropriate timing of this blood sampling would most likely have added more consistency in the interpretation of the aPTT results for females across studies.

In conclusion, the immune-deficient rat was found to be a suitable species for a long-term toxicity study of up to 52 weeks' duration and can be considered in cases where a model is needed for the long-term toxicity testing of human proteins found to be immunogenic in animals. Overall, N8-GP was well tolerated at doses ranging from 50 to 1200 IU/kg every fourth day, with no test item-related findings seen. PEG was not observed in the choroid plexus and, based on the absence of test item-related adverse findings evaluated by standard toxicological endpoints, the no observed adverse effect level for N8-GP in these studies was considered to be 1200 IU/kg every fourth day. These findings expand our knowledge of the N8-GP molecule, the safety of glycoPEGylation as a protraction technology, and the Rowett nude rat as a model for long-term toxicity studies. It is anticipated that extended half-life FVIII products, such as N8-GP, may modify current treatment regimens for hemophilia A in humans, enabling fewer infusions and thus decreasing the treatment-related burden.

## Figures and Tables

**Table 1:** Design and doses of the 26- and 52-week studies.

Group number	Study period				
	N8-GP (IU/kg/fourth day)	Dosing phase		Recovery phase	
		Males (*n*)	Females (*n*)	Males (*n*)	Females (*n*)
1 (control)	0	18 (21)	18 (21)	9 (12)	9 (12)
2	50	18 (21)	18 (21)	0	0
3	150	18 (21)	18 (21)	0	0
4	500	18 (21)	18 (21)	0	0
5	1200	18 (21)	18 (21)	9 (12)	9 (12)

Numbers given in () represent number of animals included in the 52-week study.

*Note*. The PEG doses in the dosed groups were as follows: Group Two: 0.001 mg PEG/kg/dose; Group Three: 0.003 mg PEG/kg/dose; Group Four: 0.01 mg PEG/kg/dose; and Group Five: 0.024 mg PEG/kg/dose.

**Table 2:** Sampling/examination schedule.

	Before dose	Day 1	Week 13	Week 26	Week 30	Week 48	Week 52	Recovery week 2	Recovery week 12	Recovery week 26
Toxicokinetic profile		X + O		X + O			O			
Antibody analysis				X + O			O	X + O		
Biochemistry, hematology, and coagulation			X + O	*X* ^a^	O	O			O	X
Urinalysis				X			O		O	X
Ophthalmoscopy	X + O			X			O			

*Note*. X, only in 26-week study; O, only in 52-week study.

^a^In all week 26 female main study animals, blood samples for biochemistry, hematology, and coagulation were taken two to four days after last dose and not 12 hours after dose as originally planned.

Toxicokinetic profile: samples taken before dose and at 0.25, 1, 4, 8, 12, 24, 48, and 96 hours after dose administration.

**Table 3:** List of collected tissues.

Tissue and regions examined	26-week study		52-week study	
	Weight	Light microscopy	Weight	Light microscopy
Abnormalities		X		X
Adrenals	X	X	X	X
Aorta—thoracic		X		X
Brain (cerebellum, cerebrum, midbrain, and choroid plexus)	X	X	X	X
Caecum		X		X
Colon		X		X
Dosing site		X		X
Duodenum		X		X
Epididymis	X	X	X	X
Esophagus		X		X
Eyes		X		X
Femur (femorotibial joint)		a		a
Harderian glands		X		X
Heart (including auricular and ventricular regions)	X	X	X	X
Ileum		X		X
Jejunum		X		X
Kidneys	X	X	X	X
Lachrymal glands		X		X
Liver (section from two lobes)	X	X	X	X
Lungs (section from two major lobes including bronchi)	X	X	X	X
Lymph nodes—Inguinal (left and right)		X		X
Lymph nodes —Mesenteric		X		X
Optic nerves		X		X
Ovaries	X	X	X	X
Pancreas		X		X
Pituitary	X	X	X	X
Prostate	X	X	X	X
Rectum		X		X
Salivary glands				
Submandibular	X	X	X	X
Parotid		X		X
Sublingual	X	X		X
Sciatic nerves		X		X
Seminal vesicles		X		X
Skeletal muscle (M. quadriceps)		b		a
Skin with mammary glands (inguinal area)		X		X
Spinal cord (transverse and longitudinal sections at the cervical, thoracic, and lumbar levels)		X		X
Spleen	X	X	X	X
Sternum		X		X
Stomach (including Brunner's glands)		X		X
Testes	X	X	X	X
Thyroid with parathyroid	X	X	X	X
Tongue		X		X
Trachea		X		X
Ureters		X		X
Urinary bladder		X		X
Uterus with cervix		X		X
Vagina		X		X

No thymic remnants were found in any animals in either the 26- or 52-week studies.

^a^Only two sections of the tissues were examined, one in the 26-week and one in the 52-week studies.

^b^Only one section of tissue was examined in the 26-week study.

**Table:** (a) 26-Week Study

Sex	Time of death (week)	Found dead	Euthanasia for welfare reasons	Major factors contributing to death
*0 IU/kg (control)*				
Female	13		X	Combination of findings contributed to overall poor clinical condition
Female	R 7		X	Urogenital lesions
Female	R 22		X	Lymphoma

*150 IU/kg*				
Female	10		X	Alimentary tract lesions
Female	11		X	Combination of findings contributed to overall poor clinical condition
Male	26	X		Myocarditis and epicarditis
Male	26	X		No factors contributing to death identified

*500 IU/kg*				
Female	25		X	Combination of findings contributed to overall poor clinical condition

*1200 IU/kg*				
Male	13		X	Urogenital lesions
Male	21		X	Combination of findings contributed to overall poor clinical condition
Male	R 22		X	Septicemia (sequela of skin ulceration)

*Note*. R = week of 26-week recovery phase.

**Table:** (b) 52-week study

Sex	Time of death (week)	Found dead	Euthanasia for welfare reasons	Major factors contributing to death
*0 IU/kg (control)*				
Female	6		X	Combination of findings contributed to overall poor clinical condition
Male	7		X	Combination of findings contributed to overall poor clinical condition
Male	9		X	Abscess
Female	13		X	Malignant schwannoma
Male	21		X	Gastrointestinal lesions
Male	25		X	Urogenital lesions
Male	28		X	Abscess
Female	34		X	Nasal turbinate lesion
Male	40		X	Eye lesions
Male	41	X		Kidney lesions
Male	46		X	No factors contributing to death identified
Male	47		X	Urogenital lesions
Female	51		X	Sarcoma
Male	R 4		X	Abscess

*50 IU/kg*				
Female	26		X	Malignant oligodendroglioma
Male	38		X	Lymphoma
Male	40		X	Nasal turbinate lesions
Male	41		X	Gastrointestinal lesions
Male	41	X		Lymphoma
Male	42		X	Lymphoma

*150 IU/kg*				
Male	7		X	Abscess
Male	11		X	Combination of findings contributed to overall poor clinical condition
Male	34		X	Skin lesions
Male	43		X	Sarcoma
Male	43	X		Lymphoma
Male	45	X		Lymphoma
Male	49		X	Kidney lesions

*500 IU/kg*				
Female	10		X	Gastrointestinal lesions
Male	12		X	Peritonitis
Male	15		X	Joint lesions
Female	17	X		No factors contributing to death identified
Female	27		X	No factors contributing to death identified
Female	29		X	Gastrointestinal lesions
Male	42	X		No factors contributing to death identified
Female	44		X	Skin lesions
Female	45	X		Atrial thrombosis

*1200 IU/kg*				
Female	11		X	Kidney lesions
Female	11		X	Skin lesions
Female	12	X		Urogenital lesions
Female	16		X	Gastrointestinal lesions
Male	22		X	Joint lesions
Male	28	X		Abscess
Male	31		X	Skin lesions
Female	46		X	Gastrointestinal lesions
Male	R 12		X	Squamous cell sarcoma

*Note*. R = week of 12-week recovery phase.

**Table:** (a) 26 Weeks

Group	N8-GP (IU/kg)	Dosing phase, week 13		Dosing phase, week 26	
		Males	Females	Males	Females
2	50	−13^b^	+1	−3	−6^#^
3	150	−17^b^	−2	−13^b^	−14^b#^
4	500	−19^b^	−6	−22^b^	−16^b#^
5	1200	−30^b^	−13^b^	−30^b^	−2^#^

**Table:** (b) 52 weeks

Group	N8-GP (IU/kg)	Dosing phase, week 13		Dosing phase, week 30		Dosing phase, week 48	
		Males	Females	Males	Females	Males	Females
2	50	−1	−2	+9	+14	+5	−3
3	150	−7	−8^a^	−3	−6	−9	−8^a^
4	500	−27^b^	−16^b^	−13^b^	−18^b^	−29^b^	−13^a^
5	1200	−38^b^	−26^b^	−29^b^	−27^b^	−33^b^	−26^a^

^a^ *P*< 0.05; ^b^*P*< 0.01;  ^#^In all female 26-week main study animals, blood samples were taken two to four days after last dose and not 12 hours after dose as originally planned.

**Table 6:** Anti-N8-GP antibodies detected in animals during the dosing phase.

Group	Dose (IU/kg/fourth day)	Anti-N8-GP antibodies (positive/total)		
		26-week study (week 26)	52-week study (week 26)	52-week study (week 52)
2	50	2/36	3/42	2/36
3	150	1/34	1/40	0/35
4	500	2/35	0/38	1/34
5	1200	0/34	2/39	1/35
